# The Differences in Health Care Utilization between Medical Aid and Health Insurance: A Longitudinal Study Using Propensity Score Matching

**DOI:** 10.1371/journal.pone.0119939

**Published:** 2015-03-27

**Authors:** Jae-Hyun Kim, Kwang-Soo Lee, Ki-Bong Yoo, Eun-Cheol Park

**Affiliations:** 1 Department of Public Health, Graduate School, Yonsei University, Seoul, South Korea; 2 Institute of Health Services Research, Yonsei University, Seoul, South Korea; 3 Department of Health Administration, College of Health Sciences, Yonsei University, Wonju, Gwangwondo, South Korea; 4 Department of Healthcare Management, Eulji University, Sungnam, Korea; 5 Department of Preventive Medicine, Yonsei University College of Medicine, Seoul, South Korea; Public Health Agency of Barcelona, SPAIN

## Abstract

**Study Objectives:**

Health care utilization has progressively increased, especially among Medical Aid beneficiaries in South Korea. The Medical Aid classifies beneficiaries into two categories, type 1 and 2, on the basis of being incapable (those under 18 or over 65 years of age, or disabled) or capable of working, respectively. Medical Aid has a high possibility for health care utilization due to high coverage level. In South Korea, the national health insurance (NHI) achieved very short time to establish coverage for the entire Korean population. However there there remaine a number of problems to be solved. Therefore, the objective of this study was to investigate the differences in health care utilization between Medical Aid beneficiaries and Health Insurance beneficiaries.

**Methods & Design:**

Data were collected from the Korean Welfare Panel Study from 2008 to 2012 using propensity score matching. Of the 2,316 research subjects, 579 had Medical Aid and 1,737 had health insurance. We also analyzed three dependent variables: days spent in the hospital, number of outpatient visits, and hospitalizations per year. Analysis of variance and longitudinal data analysis were used.

**Results:**

The number of outpatient visits was 1.431 times higher (p<0.0001) in Medical Aid beneficiaries, the number of hospitalizations per year was 1.604 times higher (p<0.0001) in Medical Aid beneficiaries, and the number of days spent in the hospital per year was 1.282 times higher (p<0.268) for Medical Aid beneficiaries than in individuals with Health Insurance. Medical Aid patients had a 0.874 times lower frequency of having an unmet needs due to economic barrier (95% confidence interval: 0.662-1.156).

**Conclusions:**

Health insurance coverage has an impact on health care utilization. More health care utilization among Medical Aid beneficiaries appears to have a high possibility of a moral hazard risk under the Health Insurance program. Therefore, the moral hazard for Medical Aid beneficiaries should be avoided.

## Introduction

The national health insurance (NHI) system was introduced in South Korea in 1977 and it covered the entire population in July 1989 [1].

**Fig 1:**
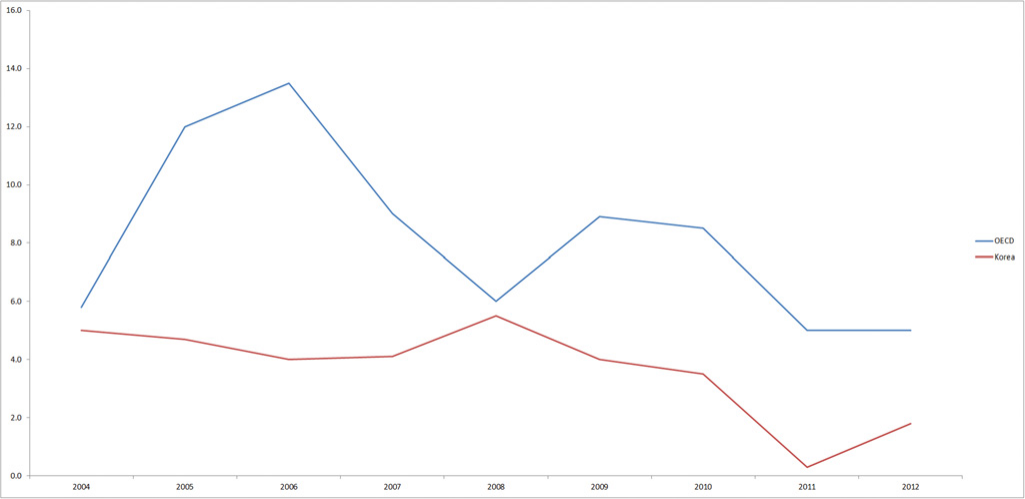
Health expenditure growth rates since 2004, Korea and OECD average.

Even though South Korea achieved universal coverage through the mandatory NHI program, public expenditure spent on NHI program as a proportion of total health expenditure was only 59%. This is in contrast to an average of 77% among member countries of the Organization for Economic Co-operation and Development (OECD) [2]. Moreover, South Korea faces the highest rate of growth in health spending, which is more than twice that of the OECD average (Fig. 1) [2,3].

One of the reasons for these phenomena is a low contribution rate to the NHI (5.89% of payroll income, 2013), which was induced from a low economic level in South Korea when the NHI started back in 1977, in addition to low contribution rates for developing non-covered services from medical care providers [4]. High out-of-pocket expenses result from copayment for insured services, full payment for non-covered services, and health care provider reimbursement for fee-for-service payments [2]. Thus, out-of-pocket expenses of individual beneficiaries have increased in spite of some efforts of expanding benefit coverage from the government, and the medical practice patterns are distorted because of the disproportionate expansion of non-covered services. As a result, benefits are relatively low and public funding is limited, leaving beneficiaries with relatively high co-payments [2].

Approximately 96% of South Korea’s population was covered by the NHI in 2007. The remaining 4% were covered by a separate program called Medical Aid, which is a public assistance program targeted at poor individuals who are recipients of the National Basic Livelihood Security System in South Korea as a part of the social welfare programs [5]. As of December 2009, Medical Aid beneficiaries represented about 3.8% of the country’s population [5]. The Medical Aid program classifies beneficiaries into two categories, type 1 and 2, on the basis of being incapable (those under 18 or over 65 years of age, or disabled) or capable of working, respectively. The cost of Medical Aid payments accounts for 16.9% of the total NHI expenditure, and has increased by an average of 15.9% year-to-year in the period of 2002 to 2006 [6]. Similarly, the number of Medical Aid beneficiaries increased by 4.5% in the 2005 to 2008 period. The national expenditure for the Medical Aid beneficiaries increased by 71.7% in the same period in South Korea, indicating that the rapid increase of Medical Aid expenditure may threaten other social welfare programs [5]. Therefore, the objective of this study was to investigate the differences in health care utilization among individuals who are covered by Medical Aid and Health Insurance.

## Methods

### Study Sample

The study data used Korean Welfare Panel Study (KOWEPS) from 2008 to 2012. Conducted by the Korea Institute for Health and Social Affairs and Seoul National University, the KOWEPS is an annual longitudinal panel survey that began in 2006 that uses proportional systematic stratified cluster sampling to select a representative sample of households in South Korea as a whole. Multiple interviews were conducted in the same households, so all members of the household 15 years or older completed the individual questionnaire when possible.

The panel consisted of 18,856 individuals from a national probability sample of 7,072 households residing in South Korea who have been surveyed annually since 2006. The sample was selected by using systematic two-stage stratified cluster sampling from 2005 census data. The KOWEPS included post-stratification weights based on 2005 census data; it was weighted by a primary sampling unit and for the intentional oversampling of low-income households. Further details of the sampling design, methods, and data sets can be found elsewhere (http://www.koweps.re.kr/). No ethical approval was needed because data for the current study are publicly available.

Respondent samples were collected from a total of 16,613 individuals from 6,314 households (wave 3), 16,255 individuals from 6,207 households (wave 4), 15,625 individuals from 6,207 households (wave 5), 14,696 individuals from 6,034 households (wave 6), and 14,604 individuals from 5,735 households (wave 7).

After applying propensity score matching method adjusting for socioeconomic status (gender, age, residential region, marital status, education level, economic activity status and ordinary income), number of chronic disease, unmet need experience. After applying propensity score matching method adjusting for income and number of chronic disease, of the 2,072 research subjects included in our study, there were 518 Medical Aid beneficiaries and 1,554 Health Insurance beneficiaries from 2008 to 2012, respectively.

### Study Variables

To investigate the difference in health care utilization between Medical Aid beneficiaries and Health Insurance beneficiaries, our target population consisted of those who had no health insurance status change for 5 years among both Health Insurance beneficiaries and Medical Aid beneficiaries. We extracted a study sample using a 1:3 propensity score adjusting for the number of chronic diseases and ordinary income by health insurance status in wave 3 (2008), and then merged individuals from wave 4 (2009) to wave 7 (2012). In this study, we analyzed three dependent variables: days spent in the hospital, number of outpatient visits, and hospitalizations per year through longitudinal data analysis using negative binomial function because health care utilization had skewed distribution. We also used repeated-measurement model (GLIMMIX model) and it determine whether probability of health care utilization changed over time, annually.

Age, gender, education level, residential region, marital status, economic activity status, number of chronic diseases, and unmet needs due to economic barrier and duration of medical treatment were included in the analyses as covariates. Education level was categorized into four groups: elementary school or lower, middle school, high school, and college or higher. Residential regions were categorized as urban (Seoul, Daejeon, Daegu, Busan, Incheon, Kwangju, or Ulsan) or rural (not classified as a city). Economic activity status was divided into two categories: yes (White colar, Blue colar) or no (housewife, students). and no (including housewives and students). Individuals were classified as married or single; the latter group included the previously married, the widowed, and the divorced. The number of chronic diseases and perceived health status were also included in our models. The number of chronic diseases was operationalized into three different categories: 0 1 and ≥2.

Self-reported data regarding unmet needs due to economic barrier was extracted from the response to the question: “Have you ever experienced not going to the hospital due to having no money during the year?” An unmet needs due to economic barrier was categorized as either “yes” or “no.”

### Statistical Analysis

Analysis of variance and longitudinal data analysis were used to investigate the association between health insurance status and health care utilization (i.e. number of days spent in the hospital, outpatient visits, and hospitalizations per year).

We conducted a longitudinal data analysis through Proc genmod procedure with the negative binomial function to investigate the number of outpatient visits and hospitalizations per year and days spent in the hospital per year because health care utilization variables had skewed distribution. In addition, we also ran a generalized linear mixed model with the binary distribution to investigate the association between unmet needs due to economic barrier and health insurance coverage.

Generalized linear models apply when the data are uncorrelated. In many studies, observations exhibit some form of dependency. For example, measurements of different attributes are taken from the same patient, observations are collected over time, sampling or randomization is carried out hiearchically, and so forth.

The models fit by the GLIMMIX procedure extend the GLM by incorporating correlations among the responses. This can be accomplished by including random effects in the linear predictor and/or by modeling the correlations among the data directly. The GLIMMIX procedure distinguishes the two approaches as “G-side” and “R-side” random effects.

This terminology draws on a common specification of the linear mixed model, the dependent variable at baseline,

*type of insurance* is the dependent variables

*β*_0_ is the intercept

*X_i_* is the covariates

*e_i_* is the error term

*i* represents each subject

This terminology draws on a common specification of the linear mixed model,

$$Y_{it}\;=\;(\beta_{0}+\mu_{i})+\;\beta_{1}{\times type\;of\;insurance}_{it}+X_{it}+e_{it}$$

where *Y*_*it*_ is the dependent variable (i.e., Number of outpatient visits, number of hospitalizations, days spent in hospital) during a time period *t* for unit *i*.

*Y* is the dependent variables

*β*_0_ is the intercept

*μ_i_* is the random effects error term

*type of insurance* is the type of insurance

*X* is the covariates

*e_it_* is the error term

*i* represents each subject

*t* represents time period

The criterion for significance was p≤0.05, two-tailed. All analyses were conducted using the SAS statistical software package version 9.2 (SAS Institute Inc., Cary, NC, USA).

### Propensity score matching

Propensity score matching (PSM) is a statistical matching technique that is being used in observational studies to reduce bias. We performs a 1:3 case-control match on the propensity score and it makes best matches first and next-best matches next, in a hierarchical sequence until no more matches can be made (nearest neighbor matching). SAS LOGISTIC procedure code was used to create the propensity score [7].

## Results

Table 1 shows the number of observation included in this study, annually and Table 2 lists the general characteristics of the subjects at baseline (2008) before propensity matching. Table 3 shows the general characteristics of the subjects at baseline (2008) after propensity matching adjusting for all variables included. Of the 2,072 research subjects, 1,554 (75.0%) had Health Insurance benefits and 518 (25.0%) received Medical Aid benefits. In the Medical Aid group the mean number of outpatient visits was 28.91 [standard deviation (SD): 40.57] per year, the mean number of hospitalizations was 0.23 (SD: 0.7) per year, and the mean number of days spent in the hospital was 3.66 (SD: 16.04) per year. In the Health Insurance group, the mean number of outpatient visits was 21.88 (SD: 32.92) per year, the mean number of hospitalizations was 0.15 (SD: 0.50) per year, and the mean number of days spent in the hospital was 3.22 (SD: 18.28) per year.

**Table 1:** Number of observations included in this study.

	Number of individuals	Number of households	Number of observations included
**2008 (wave 3)**	16,613	6,314	2,072 (Medical Aid: 518; Health insurance: 1,554)
**2009 (wave 4)**	16,255	6,207	2,072 (Medical Aid: 518; Health insurance: 1,554)
**2010 (wave 5)**	15,625	6,207	2,072 (Medical Aid: 518; Health insurance: 1,554)
**2011 (wave 6)**	14,696	6,034	2,072 (Medical Aid: 518; Health insurance: 1,554)
**2012 (wave 7)**	14,604	5,735	2,072 (Medical Aid: 518; Health insurance: 1,554)

**Table 2:** General characteristics of the study subjects at the baseline (2008) before propensity matching method.

	Total		Medical Aid		Health insurance		P-value
	Mean	SD	Mean	SD	Mean	SD	
**Number of outpatient visit**	14.23	25.22	28.25	39.31	13.53	24.10	<.0001
**Number of hospitalization**	0.13	0.52	0.23	0.68	0.12	0.51	0.000
**Days spent in hospital**	1.80	10.74	3.49	15.38	1.72	10.45	0.007
	N	%	N	%	N	%	P-value
**Gender**							0.001
** Male**	5,495	45.3	220	4.0	5,275	96.0	
** Female**	6,643	54.7	356	5.4	6,287	94.6	
**Age**							<.0001
** ≤ 19**	2,675	22.0	125	4.7	2,550	95.3	
** 20–39**	2,735	22.5	42	1.5	2,693	98.5	
** 40–59**	3,125	25.8	154	4.9	2,971	95.1	
** ≥ 60**	3,603	29.7	255	7.1	3,348	92.9	
**Residential region**							<.0001
** Urban**	5,199	42.8	301	5.8	4,898	94.2	
** Rural**	6,939	57.2	275	4.0	6,664	96.0	
**Marital status**							<.0001
** Married**	6,611	54.5	189	2.9	6,422	97.1	
** Single**	5,527	45.5	387	7.0	5,140	93.0	
**Education level**							<.0001
** ≤ Elementary**	4,971	41.0	351	7.1	4,620	92.9	
** Middle school**	1,623	13.4	101	6.2	1,522	93.8	
** High school**	3,067	25.3	103	3.4	2,964	96.6	
** ≥ College**	2,477	20.4	21	0.9	2,456	99.2	
**Ordinary income**							<.0001
** Low**	4,431	36.5	546	12.3	3,885	87.7	
** Middle**	4,350	35.8	30	0.7	4,320	99.3	
** High**	3,357	27.7	-	-	3,357	100.0	
**Number of chronic disease**							<.0001
** No**	6,625	54.6	145	2.2	6,480	97.8	
** Yes**	5,513	45.4	431	7.8	5,082	92.2	
**Duration of medical treatment**							<.0001
** Nothing**	7,308	60.2	163	2.2	7,145	97.8	
**3 month or less**	423	3.5	18	4.3	405	95.7	
** 3–6 month**	152	1.3	10	6.6	142	93.4	
** 6 month or more**	4,255	35.1	385	9.1	3,870	91.0	
**Unmet need experience**							<.0001
** Yes**	285	2.4	50	17.5	235	82.5	
** No**	11,853	97.7	526	4.4	11,327	95.6	
**Economic activity status**							<.0001
** No (including housewife)**	7,073	58.3	471	6.7	6,602	93.3	
** White collar**	2,394	19.7	31	1.3	2,363	98.7	
** Blue collar**	2,671	22.0	74	2.8	2,597	97.2	
** Total**	12,138	100.0	576	100.0	11,562	100.0	

**Table 3:** General characteristics of the study subjects at the baseline (2008) after propensity matching method.

	Total		Medical Aid		Health insurance		P-value
	Mean	SD	Mean	SD	Mean	SD	
**Number of outpatient visit**	23.64	35.11	28.91	40.57	21.88	32.92	0.000
**Number of hospitalization**	0.17	0.56	0.23	0.70	0.15	0.50	0.012
**Days spent in hospital**	3.33	17.75	3.66	16.04	3.22	18.28	0.626
	N	%	N	%	N	%	P-value
**Gender**							1.000
** Male**	768	37.1	192	25.0	576	75.0	
** Female**	1,304	62.9	326	25.0	978	75.0	
**Age**							<.0001
** ≤ 19**	478	23.1	98	20.5	380	79.5	
** 20–39**	142	6.9	31	21.8	111	78.2	
** 40–59**	419	20.2	144	34.4	275	65.6	
** ≥ 60**	1,033	49.9	245	23.7	788	76.3	
**Residential region**							0.374
** Urban**	993	47.9	257	25.9	736	74.1	
** Rural**	1,079	52.1	261	24.2	818	75.8	
**Marital status**							0.188
** Married**	1,377	66.5	332	24.1	1,045	75.9	
** Single**	695	33.5	186	26.8	509	73.2	
**Education level**							0.212
** ≤ Elementary**	1,327	64.0	322	24.3	1,005	75.7	
** Middle school**	316	15.3	87	27.5	229	72.5	
** High school**	324	15.6	89	27.5	235	72.5	
** ≥ College**	105	5.1	20	19.1	85	81.0	
**Ordinary income**							0.914
** Low**	1,950	94.1	488	25.0	1,462	75.0	
** Middle**	122	5.9	30	24.6	92	75.4	
** High**	-	-	-	-	-	-	
**Number of chronic disease**							0.061
** No**	628	30.3	140	22.3	488	77.7	
** Yes**	1,444	69.7	378	26.2	1,066	73.8	
**Duration of medical treatment**							0.367
** Nothing**	692	33.4	158	22.8	534	77.2	
** 3 month or less**	57	2.8	15	26.3	42	73.7	
** 3–6 month**	21	1.0	4	19.1	17	81.0	
** 6 month or more**	1,302	62.8	341	26.2	961	73.8	
**Unmet need experience**							0.584
** Yes**	118	5.7	32	27.1	86	72.9	
** No**	1,954	94.3	486	24.9	1,468	75.1	
**Economic activity status**							0.239
** No (including housewife)**	1,623	78.3	414	25.5	1,209	74.5	
** White collar**	155	7.5	30	19.4	125	80.7	
** Blue collar**	294	14.2	74	25.2	220	74.8	
** Total**	2,072	100.0	518	25.0	1,554	75.0	

Table 4 shows the association between variables and health care utilization from 2008. The mean number of outpatient visits for Health Insurance beneficiaries was 23.64 (SD: 35.11) per year. The mean number of outpatient visits for Medical Aid beneficiaries was 28.91 (SD: 40.57) per year. The mean number of hospitalizations for Health Insurance beneficiaries was 0.15 (SD: 0.50) per year. The mean number of hospitalizations for Medical Aid beneficiaries was 0.23 (SD: 0.7) per year. The mean number of days spent in the hospital for Health Insurance beneficiaries was 3.22 (SD: 18.28) per year. The mean number of days spent in the hospital for Medical Aid beneficiaries was 3.66 (SD: 16.04) per year.

**Table 4:** Association between variables and health care utilization at baseline (2008).

		Number of outpatient visit		P-value	Number of hospitalization		P-value	Days spent in hospital		P-value
	N	Mean	SD		Mean	SD		Mean	SD	
**Medical Security**				<.0001			0.003			0.626
** Health Insurance**	1,554	21.88	32.92		0.15	0.50		3.22	18.28	
** Medical Aid**	518	28.91	40.57		0.23	0.70		3.66	16.04	
**Gender**				<.0001			0.757			0.004
** Male**	768	17.76	29.70		0.18	0.57		4.79	26.16	
** Female**	1,304	27.10	37.52		0.17	0.55		2.47	9.78	
**Age**				<.0001			<.0001			0.001
** ≤ 19**	478	8.98	16.76		0.06	0.36		0.90	7.06	
** 20–39**	142	6.72	10.61		0.12	0.37		7.48	39.81	
** 40–59**	419	20.12	28.71		0.20	0.68		3.34	17.53	
** ≥ 60**	1,033	34.17	41.71		0.22	0.59		3.88	16.20	
**Residential region**				0.023			0.446			0.899
** Urban**	993	21.81	30.97		0.16	0.52		3.28	17.86	
** Rural**	1,079	25.32	38.47		0.18	0.59		3.38	17.65	
**Marital status**				0.061			0.149			0.011
** Married**	695	25.68	36.26		0.20	0.55		4.73	25.31	
** Single**	1,377	22.61	34.49		0.16	0.56		2.63	12.23	
**Education level**				<.0001			0.728			0.626
** ≤ Elementary**	1,327	28.17	38.89		0.18	0.59		2.98	11.53	
** Middle school**	316	17.84	29.74		0.16	0.46		3.57	25.96	
** High school**	324	14.97	23.07		0.14	0.54		4.25	24.83	
** ≥ College**	105	10.56	12.87		0.16	0.50		4.28	24.80	
**Ordinary income**				0.004			0.0036			0.7422
** Low**	1,950	24.20	35.37		0.17	0.56		3.36	17.98	
** Middle**	122	14.66	29.41		0.12	0.42		2.82	13.49	
** High**	**-**	-	-		-	-		-	-	
**Number of chronic disease**				<.0001			<.0001			<.0001
** No**	628	5.25	8.25		0.02	0.14		0.32	4.06	
** Yes**	1,444	31.63	39.10		0.24	0.65		4.64	20.96	
**Duration of medical treatment**				<.0001			<.0001			0.0002
** Nothing**	692	5.89	9.39		0.04	0.21		0.88	10.52	
** 3 month or less**	57	13.54	12.18		0.23	0.66		3.86	17.22	
** 3–6 month**	21	34.29	33.73		0.24	0.44		4.24	9.86	
** 6 month or more**	1,302	33.34	40.30		0.24	0.66		4.60	20.58	
**Economic activity status**				0.121			0.237			0.045
** No (including housewife)**	1,623	24.35	35.37		0.18	0.60		3.84	19.79	
** White collar**	155	18.65	31.81		0.13	0.36		2.01	7.74	
** Blue collar**	294	22.32	35.18		0.13	0.39		1.25	4.35	
**Unmet need experience**				0.245			0.132			0.507
** Yes**	118	27.29	42.53		0.25	0.55		2.28	7.11	
** No**	1,954	23.42	34.61		0.17	0.56		3.40	18.19	
** Total**	2,072	23.64	35.11		0.17	0.56		3.33	17.75	

The number of outpatient visits per year was 1.431 times higher (p<0.0001) for Medical Aid beneficiaries than for individuals with Health Insurance. The number of hospitalizations per year was 1.604 times higher (p<0.0001) for Medical Aid beneficiaries than for individuals with Health Insurance. The number of days spent in the hospital per year was 1.282 times higher (p<0.268) for Medical Aid beneficiaries than for individuals with Health Insurance (Table 5). Table 6 shows the adjusted effect of subgroup analysis according to economic activety status.

**Table 5:** Adjusted effect of study variables on health care utilization.

	Number of outpatient visit				Number of hospitalization				Days spent in hospital			
	RR	95% CI		P-value	RR	95% CI		P-value	RR	95% CI		P-value
**Medical Security**												
** Health Insurance**	1.000				1.000				1.000			
** Medical Aid**	1.431	1.225	1.672	<.0001	1.604	1.268	2.030	<.0001	1.282	0.826	1.991	0.268
**Gender**												
** Male**	1.000				1.000				1.000			
** Female**	1.202	1.053	1.373	0.007	0.836	0.690	1.013	0.068	0.407	0.267	0.621	<.0001
**Age**												
** ≤ 19**	1.000				1.000				1.000			
** 20–39**	0.797	0.578	1.100	0.168	1.229	0.668	2.262	0.507	3.923	1.403	10.970	0.009
** 40–59**	1.367	1.050	1.780	0.020	2.186	1.418	3.371	0.000	4.898	2.038	11.775	0.000
** ≥ 60**	1.584	1.291	1.944	<.0001	2.076	1.348	3.198	0.001	4.451	1.641	12.072	0.003
**Residential region**												
** Urban**	1.103	0.959	1.267	0.170	0.789	0.644	0.967	0.022	0.734	0.517	1.042	0.084
** Rural**	1.000				1.000				1.000			
**Marital status**												
** Married**	0.951	0.810	1.117	0.541	0.910	0.731	1.131	0.395	1.021	0.666	1.567	0.923
** Single**	1.000				1.000				1.000			
**Education level**												
** ≤ Elementary**	1.405	0.968	2.041	0.074	0.807	0.479	1.359	0.420	2.407	1.113	5.206	0.026
** Middle school**	1.066	0.727	1.563	0.742	0.764	0.438	1.332	0.343	2.229	0.980	5.071	0.056
** High school**	0.952	0.660	1.372	0.791	0.579	0.337	0.993	0.047	1.302	0.590	2.870	0.513
** ≥ College**	1.000				1.000				1.000			
**Ordinary income**												
** Low**	1.163	0.930	1.454	0.185	1.614	1.008	2.586	0.046	1.880	0.942	3.752	0.073
** Middle**	1.161	0.953	1.414	0.139	1.886	1.178	3.017	0.008	2.974	1.602	5.523	0.001
** High**	1.000				1.000				1.000			
**Number of chronic disease**												
** No**	1.000				1.000				1.000			
** Yes**	2.246	1.833	2.752	<.0001	6.683	4.591	9.728	<.0001	12.785	6.357	25.716	<.0001
**Duration of medical treatment**												
** Nothing**	1.000				1.000				1.000			
** 3 month or less**	0.933	0.735	1.184	0.567	0.638	0.404	1.008	0.054	0.421	0.176	1.012	0.053
** 3–6 month**	1.976	1.196	3.264	0.008	0.878	0.548	1.407	0.589	0.516	0.211	1.261	0.147
** 6 month or more**	1.789	1.464	2.186	<.0001	0.641	0.457	0.900	0.010	0.649	0.288	1.464	0.298
**Economic activity status**												
** No (including housewife)**	1.149	0.962	1.372	0.125	1.810	1.427	2.297	<.0001	3.810	2.302	6.309	<.0001
** White collar**	1.099	0.839	1.439	0.493	1.393	0.924	2.100	0.114	1.893	1.012	3.543	0.046
** Blue collar**	1.000				1.000				1.000			
**Unmet need experience**												
** Yes**	1.357	1.052	1.752	0.019	1.173	0.804	1.711	0.409	1.112	0.437	2.831	0.824
** No**	1.000				1.000				1.000			
**Year**												
** 2008**	1.000				1.000				1.000			
** 2009**	0.991	0.884	1.112	0.882	1.275	1.036	1.570	0.022	1.174	0.772	1.785	0.453
** 2010**	1.205	1.061	1.368	0.004	1.176	0.967	1.431	0.104	1.608	1.030	2.512	0.037
** 2011**	1.170	1.025	1.334	0.020	1.058	0.847	1.322	0.619	1.282	0.801	2.051	0.300
** 2012**	1.100	0.976	1.239	0.119	1.229	0.992	1.524	0.060	1.371	0.817	2.300	0.233

**Table 6:** Adjusted effect of subgroup analysis according to economic activity status.

	Number of outpatient visit				Number of hospitalization				Days spent in hospital			
	RR	95% CI		P-value	RR	95% CI		P-value	RR	95% CI		P-value
**Medical Security (No)**												
** Health Insurance**	ref				ref				ref			
** Medical Aid**	1.399	1.184	1.653	<.0001	1.612	1.231	2.110	0.001	1.296	0.764	2.199	0.336
**Medical Security (White collar)**												
** Health Insurance**	ref				ref				ref			
** Medical Aid**	1.518	0.849	2.715	0.159	1.473	0.757	2.866	0.254	0.738	0.234	2.323	0.603
**Medical Security (Blue collar)**												
** Health Insurance**	ref				ref				ref			
** Medical Aid**	1.252	0.892	1.758	0.193	1.206	0.709	2.053	0.490	1.225	0.590	2.545	0.586

Adjusted for gender, age, residential region, marital status, education level, ordinary income, number of chronic disease, duration of medical treatment, unmet need experience and year

Additionally, we analyzed whether an unmet needs due to economic barrier for Medical Aid beneficiaries existed (Appendix 1). The odds of an unmet needs due to economic barrier for Medical Aid beneficiaries was 0.875 times lower (95% confidence interval: 0.662–1.156) compared with Health Insurance beneficiaries.

## Discussion

The results of this study show that Medical Aid beneficiaries tended to have an increased number of outpatient visits, hospitalizations, and days spent in hospitals compared to Health Insurance beneficiaries who have relatively lower medical service coverage. Additionally, our results seem to indicate that an unmet needs due to economic barrier for Medical Aid beneficiaries on medical care utilization does not exist. These associations were independent of sociodemographic variables (age, gender, residential region, marital status, education level, and employment status), health status variables (number of chronic diseases), unmet needs due to economic barrier, and year.

Medical Aid in South Korea was started in 1977 as a way to serve the underprivileged population [5]. However, beneficiaries of Medical Aid increased concurrent to national expenditures for the program [5]. The characteristics of health care utilization by Medical Aid beneficiaries also revealed that the per capita cost of health care services for individual beneficiaries was 3.62 times that of NHI policyholders [5].

Health expenditure per capita (US dollar) in South Korea was last measured at 1438.78 in 2010 and 1702.58 in 2012, according to the World Bank and has gradually increased [8].

The Korean government implemented a copayment scheme in July 1, 2007 to address the growing cost of Medical Aid beneficiaries, whereby type 1 beneficiaries were required to pay outpatient fees of $1 (approximately 1,000 Korean Won) to primary medical institutions (Health insurance beneficiaries: 30% of total cost), $1.50 to secondary medical institutes (Health insurance beneficiaries: 35–50% of total cost), and $2 to tertiary medical institutions (Health insurance beneficiaries: 50% of total cost) [6]. As demonstrated in a previous study, it has been established that an increase in copayment leads to a reduction in the utilization of medical services [9].

In the present study, characteristics related to overuse showed chronic disease, and income. These findings are similar to those of Hadley and Holahan[10], who showed that recipients of Medicaid in the US were poorer and had more diseases and disability than those with low incomes who had private insurers. Half of those in Hadley and Holahan’s study had a cognitive disorder, and they had four times as many physical health limitations as those with low income who had private insurers.

The increase in Medical Aid expenditure may be explained by the behaviors of both recipients and providers. Specifically, two factors appear to have possibly contributed to this dramatic increase. First, free or much cheaper medical service than those covered by standard health insurance could have led recipients to visit clinics more freely [11], whereas full reimbursement for providers’ services might have led them to order frequent clinic visits and [11]. Indeed, Medical Aid expenditures of the top 2% of Medical Aid users in 2005 were 280% of the expenditure average of the total of all Medical Aid users in Korea [11]. The second have high possibility of over treatment by providers, as Medical Aid reimbursement is based on fee-for-service in South Korea [12]. Despite these facts, the government supports low-income groups who are not covered by standard health insurance.

Given this situation, it was no surprise to find the Korean government initiating strategies for rational management of over-users [13]. Underuse of medical services could render patients’ conditions worse than their current health status, which could later lead to higher long-term health care costs. This reinforces the need for education on the optimal use of Medical Aid services [12]. Medical Aid for the poor should also provide adequate medical services, but overuesr should be avoided. Beneficiaries need to learn what optimal service is and how to get it, and providers need to make appropriate decisions for the care of recipients [12].

In addition, introduction of the US case management system in 2003 was another means to curb the cost of overuse and to improve treatment outcomes and patient satisfaction [14,15]. We should also consider the integration of NHI and Medical Aid. The high spending by Medical Aid must be controlled, and there are some disparities with low income NHI beneficiaries who do not qualify for Medical Aid. Integration would solve these problems by implementing the same controlling mechanism for Medical Aid as the NHI.

Health status indicators appeared to be strong determinants of the number of physician visits and showed the expected positive and significant impact on the medical care demand. Therefore, cost sharing policies in Korean health insurance should be necessary because the relation of price and utilization of medical services in the real world should be an important factor in deciding utilization of medical services[16].

Although our additional analysis was performed as shown in Appendix 1, further studies are required for precisely identifying the possibility of unmet needs due to economic barrier of Health Insurance beneficiaries who do not qualify for Medical Aid and the possibility of moral hazards of Medical Aid beneficiaries with high health insurance coverage levels.

There are several limitations to this study. First, we were unable to analyze changes in health insurance status. Second, increasing the health care utilization of Medical Aid beneficiaries could not resolve the issue of supply-induced demand or demand-induced demand. Third, because we selected subjects with no insurance status change, there might be exist selection bias. Finally, although income and number of chronic diseases were the most important factors in determining that Health Insurance beneficiaries and Medical Aid beneficiaries were matched using propensity score matching, we could not control for other factors due to limitations of the data.

## Conclusion

There are differences in health care utilization between Medical Aid beneficiaries and Health Insurance beneficiaries. More health care utilization by Medical Aid beneficiaries appears to result in a high possibility for moral hazard under health insurance programs in response to the expanded benefits of health insurance coverage. Therefore, Medical Aid for the poor should provide adequate medical services.

### Key Message

- Medical Aid beneficiaries tended to have an increased number of outpatient visits, hospitalizations, and days spent in hospitals compared to Health Insurance beneficiaries who have relatively lower medical service coverage.
- It seems to indicate that an unmet needs due to economic barrier for Medical Aid beneficiaries on medical care utilization does not exist.
